# Modulation of Autophagy and Cell Death by Bacterial Outer-Membrane Vesicles

**DOI:** 10.3390/toxins15080502

**Published:** 2023-08-14

**Authors:** Camille Pin, Laure David, Eric Oswald

**Affiliations:** 1 IRSD, INSERM, ENVT, INRAE, Université de Toulouse, UPS, 105 Av. de Casselardit, 31300 Toulouse, France; 2 CHU Toulouse, Hôpital Purpan, Service de Bactériologie-Hygiène, Place du Docteur Baylac, 31059 Toulouse, France

**Keywords:** OMV, cell death, autophagy, pyroptosis, inflammation, apoptosis, xenophagy, infection

## Abstract

Bacteria, akin to eukaryotic cells, possess the ability to release extracellular vesicles, lipidic nanostructures that serve diverse functions in host–pathogen interactions during infections. In particular, Gram-negative bacteria produce specific vesicles with a single lipidic layer called OMVs (Outer Membrane Vesicles). These vesicles exhibit remarkable capabilities, such as disseminating throughout the entire organism, transporting toxins, and being internalized by eukaryotic cells. Notably, the cytosolic detection of lipopolysaccharides (LPSs) present at their surface initiates an immune response characterized by non-canonical inflammasome activation, resulting in pyroptotic cell death and the release of pro-inflammatory cytokines. However, the influence of these vesicles extends beyond their well-established roles, as they also profoundly impact host cell viability by directly interfering with essential cellular machinery. This comprehensive review highlights the disruptive effects of these vesicles, particularly on autophagy and associated cell death, and explores their implications for pathogen virulence during infections, as well as their potential in shaping novel therapeutic approaches.

## 1. Diversity, Biogenesis and Functions of Bacteria Extracellular Vesicles

**Figure 1:**
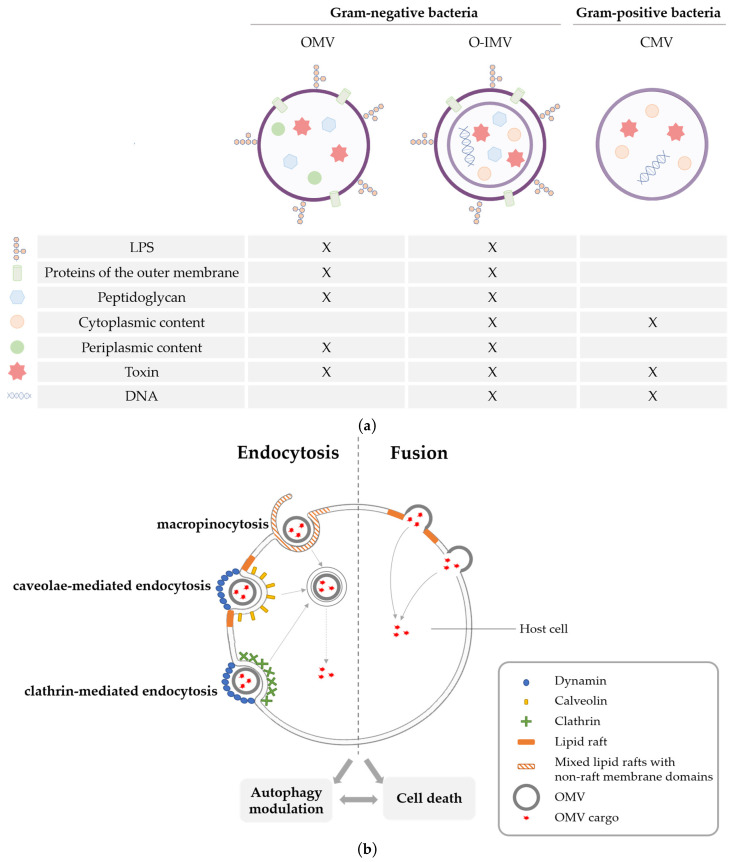
Diversity of bacteria extravesicular vesicles (**a**) composition and (**b**) mode of internalization in eukaryotic cells. (**a**) Structures and compositions of different bacterial extracellular vesicles. Outer Membrane Vesicles (OMVs) are single lipid bilayer membrane vesicles from the outer membrane of Gram-negative bacteria and carry compounds present in the outer membrane (LPSs, lipids and proteins of the outer membrane) and a content originating from the periplasmic space (fragments of peptidoglycan, secondary metabolites, proteins or toxins). Outer–Inner Membrane Vesicles (OIMVs) are double lipid bilayer membrane vesicles from the outer and the inner membrane of Gram-negative bacteria and carry periplasmic and cytoplasmic content (proteins, toxins, DNA, etc.). Cytoplasmic membrane vesicles (CMVs) from Gram-positive bacteria also contain proteins, toxins or DNA. (**b**) Internalization of BEVs in eukaryotic cells occurs by endocytosis or direct fusion at the plasma membrane. During endocytosis, the plasma membrane folds with itself and forms an endosome. This process can be lipid-raft dependent (caveolae mediated) or lipid-raft independent (clathrin mediated); both pathways depend on dynamin to close the developing endosome. Macropinocytosis consists of an actin-dependent invagination of the plasma membrane composed of both lipid rafts and non-lipid rafts structures. Fusion occurs between the membrane of the BEV and the plasma membrane, notably lipid rafts, of the eukaryotic cells. Internalization of BEVs and release of toxins into the cytoplasm of the host cell can lead to an autophagic modulation and eventually cell death (adapted from [9]).

All cells in the three domains of life are able to produce and release membrane-bound structures, called vesicles, in their extracellular medium [1]. Those vesicles are involved in cell-to-cell communication, cargo transport, stress adaptation, cell protection, survival and even pathogenicity, depending on the context. In particular, bacteria can release several types of Bacterial Extracellular Vesicles (BEVs) from their membrane. The diversity of these BEVs is firstly due to the difference in membrane structure between Gram-positive bacteria, Gram-negative bacteria and mycobacteria. Due to the structure of Gram-positive bacteria, which includes a huge layer of peptidoglycan, vesicle formation needs prior damage of peptidoglycan to release the vesicle [2]. Those vesicles, referred to here as CMVs, are formed from a curvature of the membrane and are released in the extracellular medium after the peptidoglycan degradation and sequester cytoplasmic content (Figure 1a) [3]. Vesicles produced by mycobacteria reach the extracellular space through an envelope remodeling and contain mainly cell surface and secreted proteins, but also some cytoplasmic proteins [4].

The most studied type of BEV is the Outer Membrane Vesicles (OMVs) produced by Gram-negative bacteria. Until recently, OMVs were the only type of BEVs described in Gram-negative bacteria. Pérez-Cruz et al. analyzed *Shewallena vesiculosa* vesiculation with TEM analysis and described vesicles with both outer and inner membranes, so-called O-IMVs [5]. Thus O-IMVs have a double lipidic membrane and carry periplasmic and cytoplasmic content (Figure 1a). Another study added bacterial membrane explosion (following bacterial lysis) as a mechanism for the formation of another type of BEV called explosive vesicles. These vesicles can also carry cytoplasmic content and DNA [6]. The heterogeneity of the composition of BEVs is well documented in recent reviews [7,8]. Each BEV has different biological properties. We choose to focus only on OMVs in this review. OMVs are formed through the budding of the outer membrane of the bacteria and are nanoparticles that range from 20 to 400 nm in diameter. They are composed of a single lipidic membrane containing lipopolysaccharides (LPSs) and carry a content originating from the periplasmic space. Indeed, molecules from the periplasm or the outer membrane, fragments of peptidoglycan, virulence factors, or even toxins that reach the periplasm can be sequestered and carried by those vesicles (Figure 1a).

**Figure 2:**
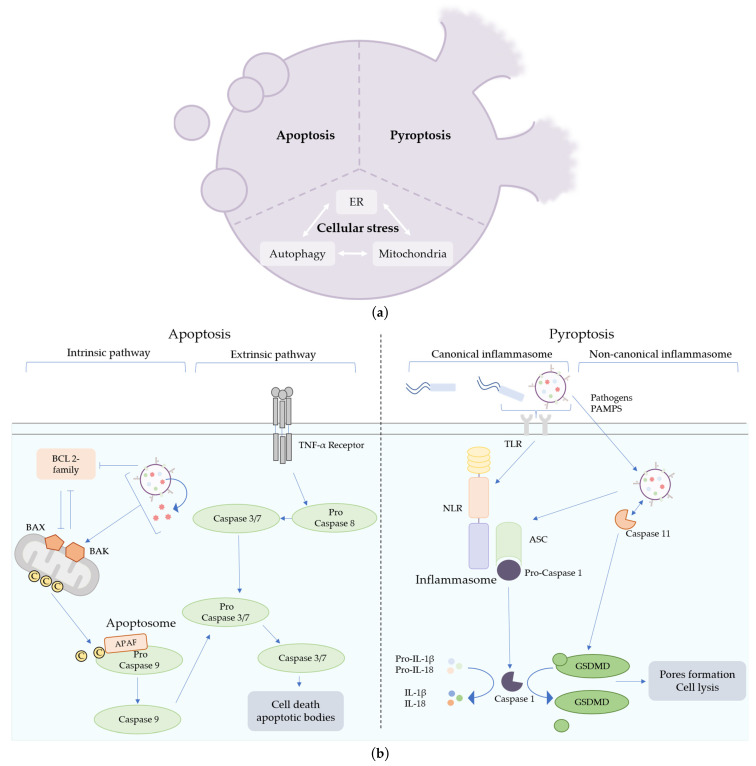
Mechanisms of cell death triggered by bacterial outer-membrane vesicles (OMVs). (**a**) Different cell death pathways can be induced by OMVs. Apoptosis is characterized by the fragmentation of the cells into apoptotic bodies, whereas pyroptosis induces pore formation in the plasma membrane of immune cells, leading to cell lysis. OMVs are also suspected to impact many types of cellular machinery (endoplasmic reticulum, autophagy or mitochondria), leading to cellular stress and cell death. (**b**) Apoptosis can be triggered through two pathways, the extrinsic and the intrinsic pathways. The extrinsic pathway is initiated by a pro-death signal from outside the cell recognized by the TNF-α receptors. The intrinsic pathway is induced by intracellular injury or activation of BAX and BAK proteins and the downregulation of pro-survival BCL 2 proteins. OMVs can trigger intrinsic apoptotic cell death by impacting mitochondrial integrity directly, by the release of toxins or by the modulation of pro-survival protein synthesis. Pyroptosis can be triggered by pathogens or pathogen-associated molecular patterns (PAMPs) recognition, leading to the activation of the canonical or the non-canonical inflammasome pathways. Caspase 11 senses LPSs or OMVs directly in the cytosol of eukaryotic cells and activates GSDMD, leading to cell lysis.

The composition of OMVs can vary according to their different biogenesis pathways. It has been demonstrated that OMVs have different structures and characteristics depending on their mode of biogenesis and the microenvironmental conditions [7,8,10]. The functions and properties of these vesicles can be influenced by the diversity of biogenesis systems, intraluminal contents or membrane composition. Indeed, OMVs are enriched in Pathogen-Associated Molecular Patterns (PAMPs), such as proteins, peptidoglycans and LPSs. PAMPs are specifically recognized by the immune system, leading to the activation of an immune response during infection, and can also trigger a pro-inflammatory cell death called pyroptosis (Figure 2a). Moreover, OMVs also have intraluminal cargo. In particular, pathogenic bacteria produce toxins, which can be secreted by OMVs and then delivered to eukaryotic cells. The internalization of these toxins in the cytoplasm leads to intracellular disorders and apoptotic cell death. Finally, phospholipids and glycolipids constituents of OMVs membranes influence host immunity [11] and could mediate other biological activities. All Gram-negative bacteria produce OMVs constitutively, but the quantity secreted varies according to environmental or intrinsic factors. As OMVs are formed from budding of the outer membrane, the membrane’s fluidity and composition impact the level of vesiculation. Differences in lipid composition between the outer membrane and OMVs of *Pseudomonas aeruginosa* suggest that OMVs are preferentially released from a specific rigid membrane domain [12]. A decrease of covalent crosslinks between the outer membrane and the peptidoglycan layer, for example, with a decrease in the number of lipoproteins (Lpps), induces an augmentation of the production of OMVs [13]. Accumulation of misfolded molecules into the periplasmic increases the release of OMVs as a mechanism to manage cellular stress [13,14]. The quorum-sensing molecule PQS has also been shown to enhance vesiculation in *Pseudomonas aeruginosa*, *Escherichia coli* (*E. coli*) and *Burkholderia cepacia* [15]. We have recently shown that HlyF, a cytoplasmic enzyme produced by certain pathogenic *E. coli* strains, enhances OMV production not only in pathogenic bacteria but also in laboratory and commensal strains [16]. The expression of HlyF is regulated by the two-component system PhoP-PhoQ, which regulates the adaptative mechanisms of bacteria during infection [17]. Thus, OMV production in bacteria expressing HlyF could be regulated, at least in part, by this two-component system to adapt to environmental conditions.

OMVs have been proposed to be the secretion system type zero (SST0) among bacteria because of their ability to carry specific cargo in their lumen [18]. Cargo can then be delivered to other bacteria and also into host cells by OMVs [18,19]. In particular, OMVs are an efficient and protective method for bacteria to deliver toxins to eukaryotic cells [20,21,22]. Indeed, OMVs can be internalized into eukaryotic cells via endocytosis or by direct fusion with the host cell membranes (Figure 1b) [9]. The size and composition of OMV membranes impact their mode of internalization. Small OMVs under 100 nm and OMVs produced by bacterial strains with an intact O antigen on their surface are internalized preferentially through caveolin-mediated endocytosis. Larger vesicles are internalized through clathrin-mediated endocytosis or macropinocytosis [11,23,24]. OMVs produced by *Pseudomonas aeruginosa* are internalized by direct fusion of OMVs with lipid rafts in the host plasma membrane. In that case, toxins present in OMVs are delivered 17,000 times more effectively into host cells than free toxins [21]. OMVs also protect toxins from degradation in the host. OMVs from *Vibrio cholerae* protect the cholera toxin contained in their lumen from proteolytic degradation in the intestinal tract, whereas free cholera toxin is degraded [25]. The diversity of OMV modes of internalization and content could explain their differences in biological activity.

Because of their nano size, OMVs have been proposed to reach host cells deep within tissues that are inaccessible to bacteria [26]. Even if most studies focus on OMV production in vitro, it has been shown that OMVs can be released in vivo in a murine pneumonia model during an infection with *Actinetobacter baumanii* [27]. Indeed OMVs have been shown to bypass the intestinal epithelial barrier in a transwell model [28]. Another study showed, with an elegant Cre-LoxP system, that OMVs produced directly in the mouse intestinal lumen can reach the heart, spleen, liver and brain, pointing out the ability of OMVs to spread in the whole organism [29]. OMVs were also found in human body fluids, notably in blood and stool [30]. The ability of OMVs to diffuse through the body and to reach cells that are inaccessible to bacteria raises the question of their contribution to bacterial pathogenicity during an infection, considering the fact that OMVs alone can have deleterious effects on eukaryotic cells.

## 2. Cell Death Pathways Triggered by OMVs

### 2.1. OMVs Induced Pyroptosis

Pyroptosis was first described nearly 20 years ago and was first referred to as caspase-1-dependent apoptosis [31] before being considered as a different type of cell death and properly named pyroptosis [32]. Pyroptosis is characterized by its inflammatory nature and by the activation of a caspase-1-dependent pathway, leading to the lysis of the cells. As a pro-inflammatory cell death, pyroptosis is induced mostly in immune cells such as macrophages and neutrophils and exists at a very low level in other cell types. Initiation of pyroptosis is triggered by specific signals of infection, such as bacteria detection by the recognition of PAMPs at their surfaces.

Pyroptosis can be activated by two signaling pathways, the canonical and the non-canonical inflammasome pathways. Both pathways end with gasdermin-D (GSDMD) activation and cleavage of pro-IL-1β and pro-IL-18 cytokines into their active form by caspase-1. Activated GSDMD forms pores in the plasma membrane, leading to cell lysis (Figure 2a), which facilitates the secretion of mature IL-1β and IL-18. These two pathways then lead to cell death and the massive release of pro-inflammatory cytokines into the extracellular space, allowing the recruitment and activation of immune cells to fight against the infection. The main difference between the canonical and the non-canonical pathways is the dependence on caspase-4/5 (or murine caspase-11) activation. In the non-canonical inflammasome pathway, caspase-4/5 senses LPSs in the cytoplasm that can directly activate GSDMD, leading to pyroptosis. In the canonical inflammasome pathway, activator signals are sensed by receptors, such as Toll-Like Receptors (TLR), located on plasma membranes. This recognition leads directly to the formation of a NOD-Like Receptor (NLR), ASC and pro-caspase-1 inflammasome complexes, which trigger the activation of caspase-1 independently of caspase-4/5 (or caspase-11) activation (Figure 2b) [33]. Differences in activator signals, such as a different pathogen during infection, impact the composition of the inflammasome and, in particular, the type of NLR. For example, NLRP3 is activated by the detection of *E. coli* or LPS recognition, whereas NLRC4 is activated by the recognition of *Pseudomonas aeruginosa* or flagellin [34,35].

LPSs, components of the membrane of OMVs, are the most potent PAMPs that can activate the non-canonical inflammasome. Many studies showed that OMVs are able to trigger pyroptosis in macrophages [36,37,38,39]. OMVs produced by Enterohaemorragic *E. coli* (EHEC) are internalized by endocytosis, but LPSs are able to detach from OMVs, leaving the endosome compartment and reaching the cytoplasm. In the cytoplasm of the host cell, LPSs then directly bind the murine caspase 11 (or human caspase 4/5) and trigger the activation of pyroptosis [36]. Another study showed that proteins GBP2 and GBP5 are indispensable for the recognition of LPSs by caspase-1 and promote it [37].

Similarly, in OMVs produced by *Bordetella pertussis*, the LPS analogs lipo-oligosaccharides (LOSs) carried by OMVs reach the cytosol after their internalization and trigger the formation of an inflammasome, leading to the activation of GSDMD and induction of pyroptosis [38]. As with toxins, OMVs are more efficient for delivering LPSs in eukaryotic cells than bacteria. A study showed that OMVs from *Porphyromonas gingivalis* induce complete activation of pyroptosis with cell death and the release of interleukin, whereas treatment with the same quantity of *Porphyromonas gingivalis* bacteria just induces the activation of the beginning of the pathway with inflammasome formation [39]. Other studies also showed that, in addition to LPS, intrinsic membrane components of OMVs, such as peptidoglycan and flagellin, participate in the activation of these pro-inflammatory signaling pathways. OMVs produced by *Helicobacter pylori*, *Pseudomonas aeruginosa* and *Neisseria gonorrhoeae* carry and deliver peptidoglycan in epithelial cells, which are detected by cytoplasmic NOD1 receptors and promote inflammation [40,41]. OMVs from *Salmonella typhimurium* and *Pseudomonas aeruginosa* are also able to transport flagellin directly into eukaryotic cells, triggering an NLRC4-dependent activation of the inflammasome [35]. 

All these studies point out the vast pyroptotic and pro-inflammatory capabilities of OMVs. Due to their intrinsic composition and regardless of their cargo, OMVs can affect the host immune system and cell viability by inducing pyroptosis. In addition, due to their mode of secretion, OMVs carry toxins or virulence factors and deliver them to the host cell. These toxins impact the activity and viability of eukaryotic cells and can lead to intracellular injury or even apoptosis.

### 2.2. OMVs Induced Apoptosis

Apoptosis was the first programmed cell death described in the 1970s [42]. Two pathways are involved in the activation of apoptosis: the extrinsic and the intrinsic pathways. Both extrinsic and intrinsic pathways activate mature caspase 3/7 through caspase 8 and caspase 9, respectively, which leads to cell death through fragmentation of cells via the formation of vesicles about 1 to 5 µm in size called apoptotic bodies (Figure 2a). The extrinsic pathway is characterized by the activation of the TNF-α receptor at the plasma membrane by pro-death molecules, which activate pro-caspase 8 (Figure 2b). The intrinsic pathway depends on mitochondria and molecules regulating its integrity. Activation of extrinsic pathways is triggered by pro-death extracellular signals, whereas activation of intrinsic pathways is related to intracellular injury. In this case, activation of the pro-apoptotic proteins BAX and BAK by downregulation of the pro-survival BCL-2 family or mitochondrial damage leads to permeabilization of the mitochondrial membrane, allowing the release of cytochrome C into the cytoplasm. Cytochrome C binds to Apoptotic peptidase factor 1 (APAF1) and pro-caspase 9. Together, these three molecules form the apoptosome and activate the pro-caspase 9 in its active form, leading to cell death (Figure 2b) [33]. 

As described before, OMVs are able to transport virulence factors or toxins and to be internalized into host cells. Some OMV-associated virulence factors were described to specifically impact mitochondria, like outer membrane proteins (Omp). OmpA and Omp38 from *Acinetobacter baumanii* are secreted through OMV-secretion. After OMV-internalization in eukaryotic cells, OmpA and Omp38 colocalize with mitochondria and compromise their membrane integrity, leading to apoptotic cell death [43,44,45,46]. OMV toxins can target mitochondria by direct or indirect delivery. Entire OMVs derived from *Neisseria gonorrhoeae* have been shown to interact with mitochondria allowing the direct delivery of the toxin PorB, known to induce cytochrome C release and apoptotic cell death [47]. EHEC hemolysin, a toxin produced by EHEC during infection that is present in OMVs, detaches from OMVs in the endosome after OMVs internalization and targets mitochondria, leading to the release of cytochrome C and apoptotic cell death [48]. In addition to EHEC hemolysin, the toxins Stx2a and CdtV present in the same OMVs were described as direct caspase 9 activators [20]. The delivery of toxins to the mitochondria by OMVs to induce apoptosis is thus a common mechanism shared by several pathogenic bacteria. In addition, a recent study also showed that OMVs derived from *Neisseria gonorrhoeae*, *Pseudomonas aeruginosa* and *E. coli* reduce mitochondrial membrane potential in a non-specific manner and activate intrinsic apoptosis by reducing the synthesis of pro-survival BCL-2, leading to activation of BAX and BAK [49].

In summary, OMVs can impact cell viability by delivering toxins or by internalization of PAMPs. Several studies showed the implication of OMVs in cell death, especially apoptosis and pyroptosis. However, OMVs are very diverse, in particular, regarding their lipid compositions of membranes. As lipids are known to have a broad spectrum of activities on eukaryotic cells [11,50], OMVs can be expected to have many other biological activities (Figure 2a). Yet few other functions, such as autophagy, have been elucidated to date concerning the role of OMVs in the pathophysiology of infections.

## 3. Modulation of Autophagy by OMVs and Consequences on Cell Fate

### 3.1. Autophagy Induction as an Antimicrobial Mechanism

**Figure 3:**
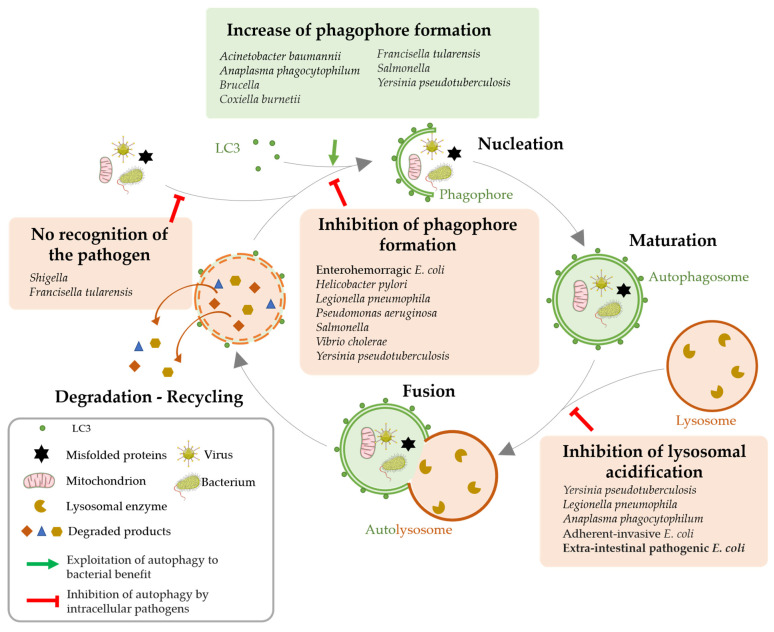
Modulation of host cell autophagy by pathogenic bacteria. The different steps of the autophagic flux and the possible bacterial subversions that lead to autophagy are depicted: (1) Increased autophagosomes formation, (2) loss of pathogen recognition, (3) inhibition of phagophore formation and (4) inhibition of lysosomal degradation (adapted from [60]).

Macroautophagy, more commonly referred to as “autophagy”, is the major intracellular pathway to the lysosome. It comprises four main steps. It begins with the induction, i.e., the formation of a double-membrane structure called a phagophore. It gradually closes in on itself, forming a closed structure called an autophagosome. During their maturation, autophagosomes encapsulate intracellular components in order to be degraded. Mature autophagosomes finally fuse with a lysosome, an acidic vesicle containing enzymes that digest the content of the resulting autolysosome (Figure 3) [51,52,53].

Autophagy can be non-selective, in which case it is associated with the indiscriminate engulfment of cytosolic components, notably in response to nutrient starvation. In contrast, selective autophagy degrades specific targets, such as damaged organelles (mitophagy, lysophagy, ER-phagy, ribophagy), aggregated proteins (aggrephagy) or invading bacteria (xenophagy) [54].

Xenophagy is the first line of defense against pathogens. It occurs in non-phagocytic cells, such as epithelial cells, that constitute the first barrier against infection. Xenophagy is induced by a lot of events associated with bacterial infection, such as the transient loss of nutrients or detection of PAMPs. The process of xenophagy involves the same steps as canonical autophagy, the specificity being that intracellular pathogens are targeted into autophagosomes [52,55,56]. In line with this idea, OMVs deliver toxins that are responsible for autophagic induction. In that case, autophagy acts as a cellular defense mechanism against OMV-associated bacterial virulence factors. *V. cholerae* produces cytolysin that is encapsulated into OMVs and delivered to host epithelial cells. Then the autophagic flux is induced in these cells as an antimicrobial response to the toxin [57]. 

But, as with bacteria, the detection of OMVs by eukaryotic cells leads to induction of autophagy as an antimicrobial mechanism due to OMV’s intrinsic composition and independent of their content. Indeed, OMVs inherently carry PAMPs, notably LPSs, which are recognized by the host cells and induce the activation of autophagy to target them for degradation. For example, *Helicobacter pylori* or *Pseudomonas aeruginosa* produce and deliver OMVs into host cells. This in turn triggers the activation of the host pattern recognition receptor (PRR) NOD1, which senses peptidoglycan present in these OMVs and ulti-mately activates the induction of autophagy in epithelial cells but not in macrophages. These results show that the consequences of the internalization of OMVs do not only depend on their composition and their cargo but also on the type of host cell [41]. A similar mechanism has also been described with *Salmonella*. Indeed, this enterobacterium produces OMVs that are recognized by host epithelial cells. This recognition leads to the activation of xenophagy through the activation of AMPK (that in turn inhibits mTOR C1 and activates ULK1, a major event in the autophagosome formation) as a protective mechanism against infection.

Interestingly, OMV-detection by eukaryotic cells can pre-activate xenophagy as a priming response in case of a future bacterial invasion to anticipate the clearance of pathogens even before their adhesion and entry into the cell. It is important to note that OMVs induce only a pre-activation of xenophagy kinases, meaning that bulk autophagy is not induced at that time, preventing indiscriminate degradation of cytoplasm, which would be energetically wasteful and would potentially lead to ineffective response to the detection of extracellular pathogens [58,59].

Because xenophagy provides the first line of innate defense and appears to be very important for intracellular pathogens clearance, many bacteria evolved anti-autophagy strategies to survive, replicate and proliferate inside cells and spread across tissues undetected and unscathed. Such strategies can involve avoidance of cellular surveillance mechanisms, impairment of phagophore formation as well as subversion of autophagy machinery (Figure 3) [56,60,61,62,63,64].

### 3.2. Autophagy Impairment for Bacterial Benefit

A common strategy used by bacterial pathogens to escape xenophagy is to shield themselves from being targeted by ubiquitin chains or being recognized by autophagy proteins [56]. *Shigella* secretes virulence protein IcsB, which competes with ATG5 to bind the bacterial membrane protein IcsA and therefore helps the pathogen avoid detection by this host autophagic protein [65]. Bacterial pathogens can also inhibit phagophore formation or autophagosome maturation. For instance, *Pseudomonas aeruginosa* is able to reside and replicate inside airway epithelial cells thanks to its ability to excrete through a type III secretion system a cytotoxin called ExoS. ExoS inhibits Vps34 activity, leading to an early inhibition of the autophagic process [66]. Finally, pathogens can prevent lysosomal fusion. Indeed, the outer membrane protein A of *Acinetobacter baumannii* prevents autophagosome and lysosome fusion, resulting in incomplete autophagy in epithelial and macrophagic cell lines [43]. *Yersinia enterocolitica* resides in autophagosomal vacuoles but is protected against acidification by actively inhibiting the formation of autolysosomes. This mechanism, associated with the active induction of the autophagic flux, allows the formation of a large number of autophagosomes that can contain bacteria and support their proliferation [67]. 

Although many bacteria have been described as being capable of modulating autophagy to their own profit (Figure 3), notably through the excretion of toxins, to date, only one study has reported on the role of OMVs in inhibiting autophagy. We established that OMVs produced by pathogenic *E. coli* expressing HlyF have unique intrinsic properties. In particular, they are able to be internalized into host cells and block the host autophagic flux—both in epithelial cells and macrophages—while not detected in autophagosomes. We verified that the intrinsic composition of these OMVs, rather than the presence of luminal cargo, was responsible for this phenotype. Moreover, we demonstrated that these OMVs blocked autophagosome maturation and acidification by impairing their fusion with lysosomes and thus inhibiting their clearance from host cytosol [68]. Thus, pathogenic bacteria expressing HlyF could exploit OMVs properties to block their autophagosomal clearance into epithelial cells and macrophages.

### 3.3. Consequences of Autophagy Modulation by Pathogenic Bacteria

Autophagy is a major negative regulator of excessive inflammation. It notably downregulates the non-canonical inflammasome activation and IL-1β release by pyroptosis, one of the main mechanisms through which innate and adaptive immune responses are induced by an infection. This negative regulation mainly relies on the effects of the secretion of immune mediators and on the removal of endogenous inflammasome agonists such as PAMPs. Therefore, inhibition of autophagosomal clearance of these inflammasome activators impairs inflammasome negative feedback control, leading to an uncontrolled inflammatory condition detrimental to the host [52,63,69,70,71,72]. In line with this idea, *Helicobacter pylori* CagA protein negatively regulates autophagy and thus exacerbates inflammation activation [73]. Pathogenic adherent-invasive *E. coli* (AIEC) are internalized into neutrophils and access to autophagosomes. There, to prevent their degradation, they are able to block lysosomal acidification, inhibiting the autophagic process. Consequently, this blockage induces the production of pro-inflammatory cytokine IL-8 and, ultimately, neutrophil non-apoptotic cell death in response to elevated sustained stress associated with autophagic blockage. On the contrary, upregulation of autophagy in infected neutrophils by nutrient starvation reduces the survival rate of AIEC and decreases the production and release of IL-8 and cell death. Therefore, in neutrophils, autophagy controls the production of inflammatory cytokines and the fate of these cells by eliminating pathogens [74]. We showed that OMVs produced by pathogenic *E. coli* expressing HlyF provoked a highly exacerbated activation of the non-canonical inflammasome, IL-1β release and massive cell death of macrophages. We showed that this overactivation was caused by OMV-associated autophagic blockade and the loss of negative feedback control [68]. This situation is deleterious for the host since the uncontrolled inflammatory burst is not efficient at fighting against infection and can progress to sepsis and threaten patients’ lives in severe cases [72,75]. OMVs could be key players in the establishment of sepsis due to their nanostructure allowing their dissemination and their pro-inflammatory properties. Indeed, the role of OMVs in sepsis is highlighted by the fact that injection of OMVs alone in mice recapitulates sepsis-associated exacerbated inflammation and mortality [76].

Thus, autophagy modulation may provide new insights into therapeutic strategies to fight against intracellular pathogens.

## 4. Concluding Remarks and Outlook

In this review, we have described the diverse capabilities of bacteria in producing various types of OMVs, which differ in their lipid, LPS, protein, and intraluminal cargo compositions, including toxins. These differences in OMVs properties are influenced by factors such as the producing bacterial strains (e.g., HlyF expression in *E. coli*) and the environmental conditions. Certain OMVs have been found to contribute to infection progression due to the presence of toxins, their nanostructure that facilitates dissemination, and their ability to be internalized by host cells. Additionally, their inherent composition, particularly the presence of LPSs on their surface, modulates host cellular mechanisms. While the roles of OMVs in pro-inflammatory and immune responses are well-known, their interaction with autophagy, which plays a crucial role in combating antimicrobial agents and intracellular pathogens, remains less understood. Indeed, we have shown that OMVs are more than just a carrier of PAMPS; the structuring of OMVs alters the properties and trafficking of these PAMPs. Our research has revealed for the first time that OMVs produced by HlyF-expressing *E. coli* significantly enhance the virulence of these strains by inhibiting autophagy in both epithelial cells and macrophages, promoting cell death. Therefore, these important results may provide new targets for the future development of host-targeted therapies complementary to the use of antibiotics therapeutic interventions. Indeed, restoring autophagic flux could counteract the detrimental effects associated with these OMVs and provide a viable alternative to antibiotic treatment, as demonstrated in the case of AIEC infection in neutrophils [74]. However, it is crucial to precisely understand the underlying mechanism that is impaired when targeting autophagy as a therapeutic approach, considering its multifaceted roles in eukaryotic cells. Manipulating autophagy may impact numerous signaling pathways or processes, resulting in diverse effects on disease outcomes [55].

On the other hand, gaining a better understanding of the mechanisms that confer specific properties to bacteria and OMVs, such as autophagy modulation, could enable their utilization in biomedical applications. Bacteria are already employed in the treatment of infections, metabolic disorders, immune or inflammatory diseases, neurodegenerative diseases, and cancers as biotherapies. However, in order to develop targeted therapeutic approaches utilizing microbial products and refine the conditions for their use, it is imperative to enhance our understanding of the specific properties of bacteria or OMVs and their interactions with the host [77,78].

## Data Availability

Not applicable.
